# Supplementary material for: Ebola virus VP35 hijacks the PKA-CREB1 pathway for replication and pathogenesis by AKIP1 association
Source: Nat Commun. 2022 Apr 26;13:2256. doi: 10.1038/s41467-022-29948-4 (PMC9042921; doi:10.1038/s41467-022-29948-4)
Supplement: Supplementary file 1 — Supplementary Information [file 41467_2022_29948_MOESM1_ESM.pdf]

Supplementary Information

Ebola Virus VP35 Hijacks the PKA-CREB1 Pathway for Replication and Pathogenesis by AKIP1 Association

Lin Zhu[1,8], Ting Gao[1,8], Yi Huang[2,8], Jing Jin[3], Di Wang[3], Leike Zhang[2], Yanwen Jin[1], Ping Li[1], Yong Hu[1], Yan Wu[2], Hainan Liu[1], Qincai Dong[1], Guangfei Wang[1], Tong Zheng[1], Caiwei Song[1], Yu Bai[3], Xun Zhang[3], Yaoning Liu[3], Weihong Yang[3], Ke Xu[4], Gang Zou[5], Lei Zhao[6], Ruiyuan Cao[6], Wu Zhong[6], Xianzhu Xia[7], Gengfu Xiao[2,*], Xuan Liu[1,*] and Cheng Cao[1,*]

[1]Beijing Institute of Biotechnology, Beijing 100850, China

[2]National Biosafety Laboratory, Chinese Academy of Sciences, Wuhan, Hubei 430020, China.

[3]Institute of Physical Science and Information Technology, Anhui University, Hefei, Anhui 230601, China.

[4]State Key Laboratory of Virology, College of Life Sciences, Wuhan University, Wuhan 430072, China.

[5]Insitut Pasteur of Shanghai, Chinese Academy of Sciences, Shanghai 200031, China.

[6]National Engineering Research Center for the Emergency Drug, Beijing Institute of Pharmacology and Toxicology, Beijing 100850, China.

[7]Changchun Veterinary Research Institute, Chinese Academy of Agricultural Sciences, Changchun 130000, China.

[8]These authors contributed equally.

[*]Correspondence to:

Cheng Cao (Phone: +86-10-66931932; E-mail: caoc@nic.bmi.ac.cn)

Xuan Liu (Phone: +86-10-66931932; E-mail: liux931932@163.com)

Gengfu Xiao (Phone: +86-27-87198685; E-mail: xiaogf@wh.iov.cn)

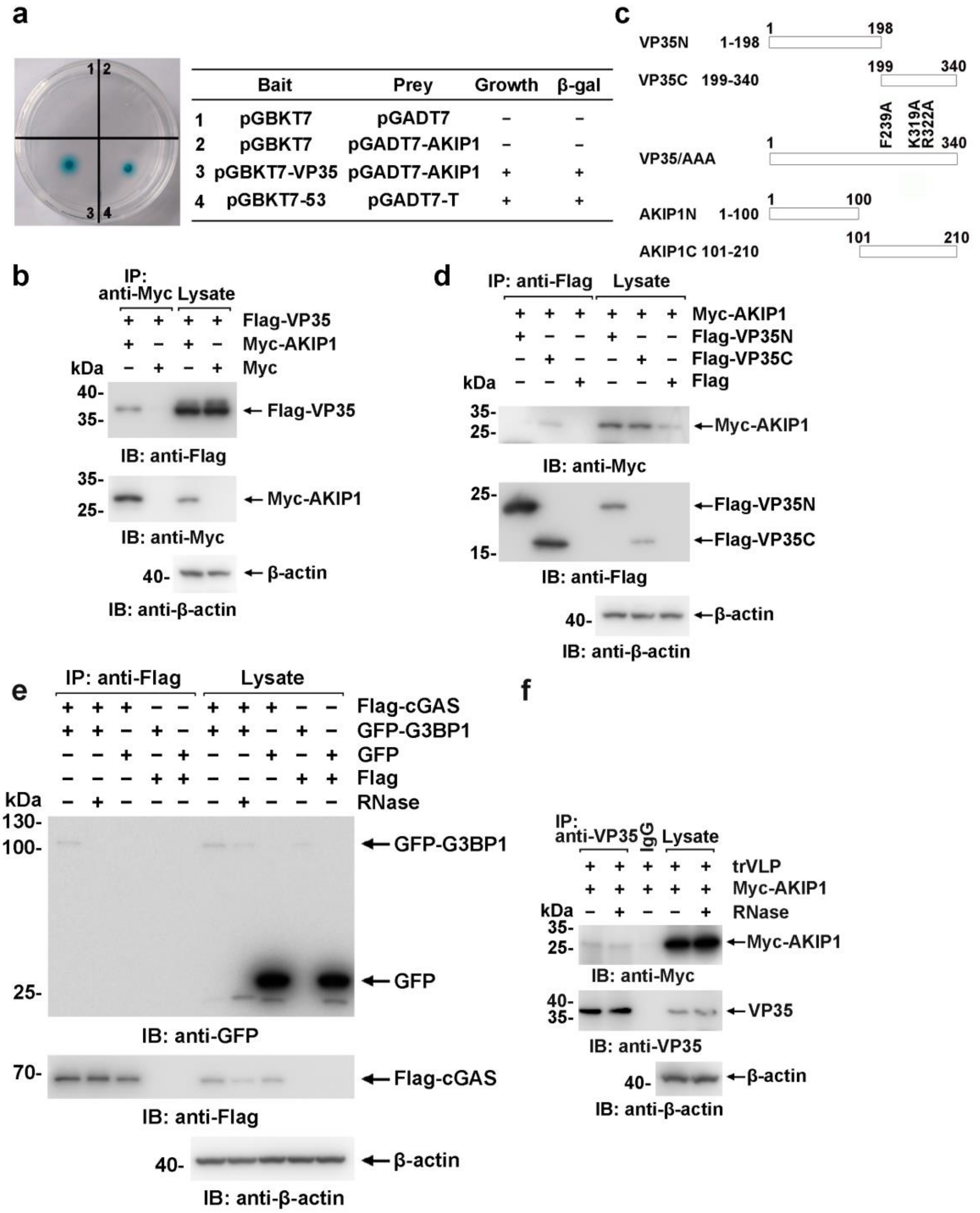

Supplementary Fig. 1 The interaction between EBOV VP35 and AKIP1.

a, Yeast Y2HGold cells were transformed with the indicated plasmids (bait and prey for the two-hybrid assay) and grown on selective medium. Positive interactions were indicated by colonies that grew on selective medium with β-galactosidase activity (blue). **b, d,** Lysates of HEK293 cells cotransfected with the indicated plasmids were analyzed by immunoprecipitation

and immunoblotting. The data from two independent experiments are presented. **c,** Graphical representation of VP35 and AKIP1 mutants. **e,** Lysates of HEK293 cells transfected with the indicated plasmids were treated with/without RNase (the mixture of RNase A and RNase T1) and analyzed using immunoprecipitation and immunoblotting. The data from three independent experiments are presented. **f,** Lysates of HEK293 cells transfected with Myc-AKIP1 and the EBOV minigenome (p0) were immunoprecipitated using anti-VP35 or IgG and analyzed by immunoblotting with indicated antibodies. The data from two independent experiments are presented.

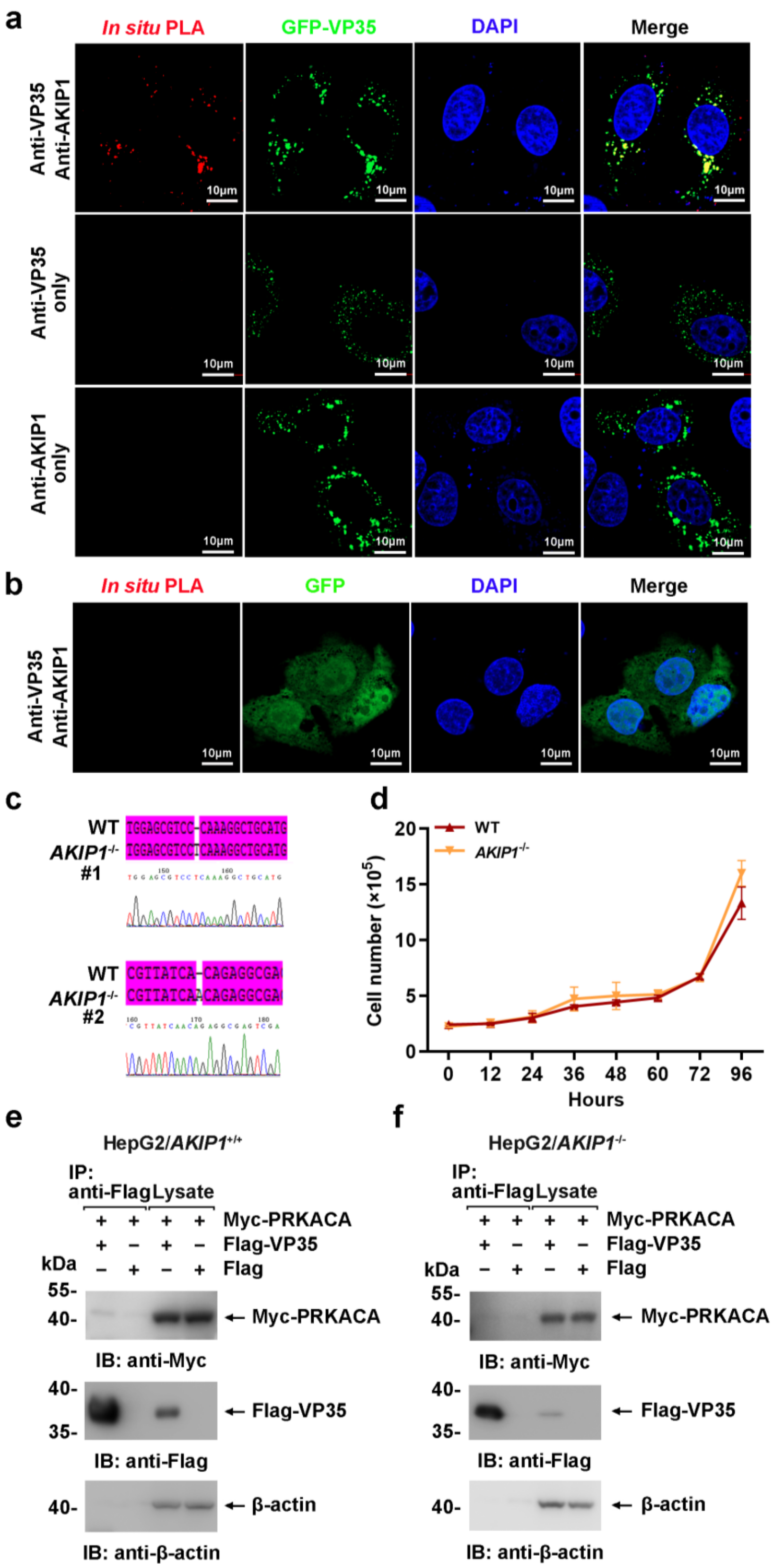

Supplementary Fig. 2 EBOV VP35 interacts with PRKACA through AKIP1.

a, b, HepG2 cells were transfected with GFP-VP35 (**a**) or the GFP vector (**b**) and then subjected to

an *in situ* PLA assay with the indicated antibodies. **c, d,** Two independent clones of *AKIP1*$^{-/-}$ HepG2 cells (KO1 and KO2) were generated with CRISPR/Cas9 and confirmed by sequencing (**c**). Cell proliferation is shown in **(d)**. The mean ± s.e.m. from three independent assays is presented (n=3). **e, f,** Lysates of WT (**e**) and *AKIP1*$^{-/-}$ (**f**) HepG2 cells cotransfected with the indicated plasmids were analyzed using immunoprecipitation and immunoblotting. At least three independent replicates of all experiments were performed.

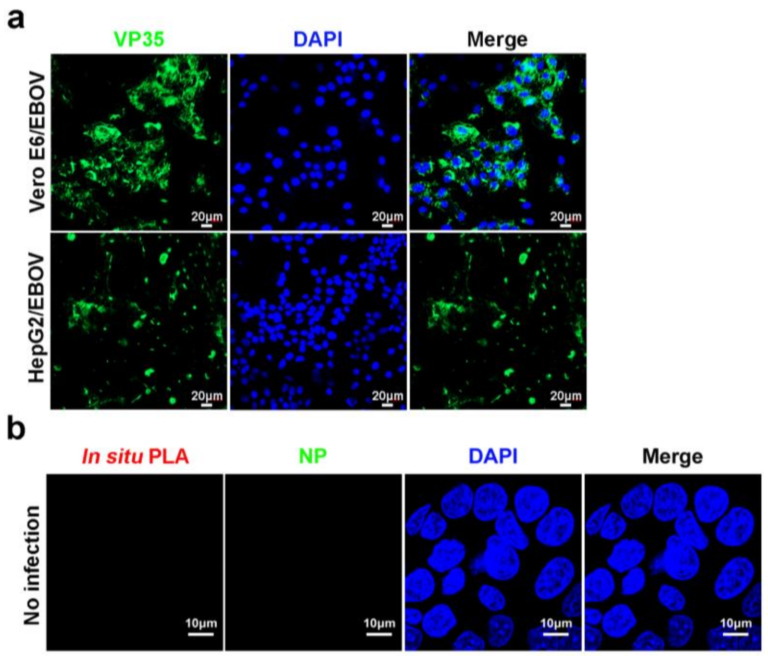

Supplementary Fig. 3 EBOV VP35 associates with AKIP1 in the cell.

a, HepG2 or Vero E6 cells were infected with live EBOV (MOI=10) for 72 h and immunostained with an anti-VP35 antibody. The data from three independent experiments are presented. **b,** As a control for the result presented in the main text (Fig. 2b), uninfected HepG2 cells were subjected to an *in situ* PLA assay and immunofluorescence staining with an anti-NP antibody. The data from three independent experiments are presented.

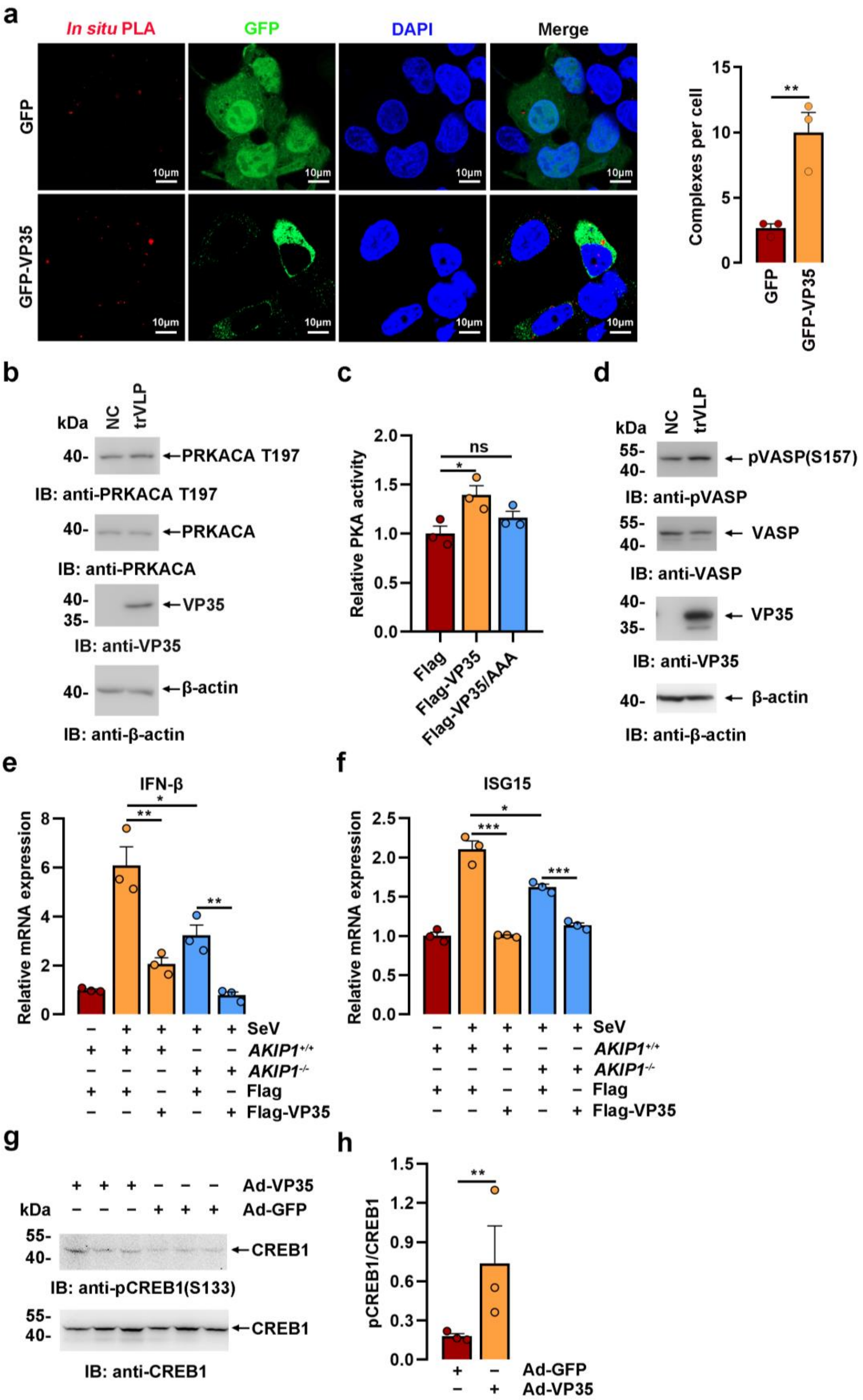

Supplementary Fig. 4 EBOV VP35 promotes the AKIP1-PKA interaction and CREB1 phosphorylation.

a, HepG2 cells transfected with GFP-VP35 or GFP were subjected to *in situ* PLA with anti-AKIP1 and anti-PRKACA antibodies (left panel), and the signal for the PLA complex in each cell was counted from at least 10 cells and presented as the means ± s.e.m. (n=10; right panel). Differences between the two groups were evaluated using the two-sided unpaired Student's *t*-test. $^{**}P < 0.01$. **b,** Lysates of HepG2 cells transfected with or without the EBOV minigenome (p0) were analyzed by immunoblotting with an anti-pPRKACA-T197 antibody. **c,** Lysates of HepG2 cells transfected with Flag vector, Flag-VP35 or Flag-VP35/AAA mutant plasmids were subjected to PKA activity assays. Differences between the two groups were evaluated using the two-sided unpaired Student's *t*-test. Data are presented as mean ± s.e.m. (ns, not significant; $^{*}P < 0.05$). **d,** HepG2 cells transfected with or without the EBOV minigenome (p0) were analyzed using immunoblotting with an anti-pVASP-S157 antibody. **e, f,** WT and *AKIP1*-depleted HepG2 cells were transfected with Flag or Flag-VP35 plasmid for 24 h, an then infected with or without SeV at an MOI of 2 for 12 h. The mRNA levels of IFN-β (**e**) and ISG15 (**f**) were quantified using qRT-PCR. Differences between the two groups were evaluated using the two-sided unpaired Student's *t*-test. Data are presented as mean ± s.e.m. ($^{*}P < 0.05$; $^{**}P < 0.01$; $^{***}P < 0.001$). **g,** C57BL/6N mice (10 weeks) were infected with Ad-VP35 or Ad-GFP (2×10^9 PFU) via the tail vein twice at 24 h intervals. Three days later, the liver tissues were homogenized and analyzed by immunoblotting with anti-CREB1 pS133 or anti-CREB1 antibodies. **h**, The ratio of CREB1 pS133/CREB1 was quantified through densitometry with ImageJ software (n=3). Differences between the two groups were evaluated using the two-sided unpaired Student's *t*-test. All data from three independent experiments are presented as the mean ± s.e.m. (**P < 0.01).

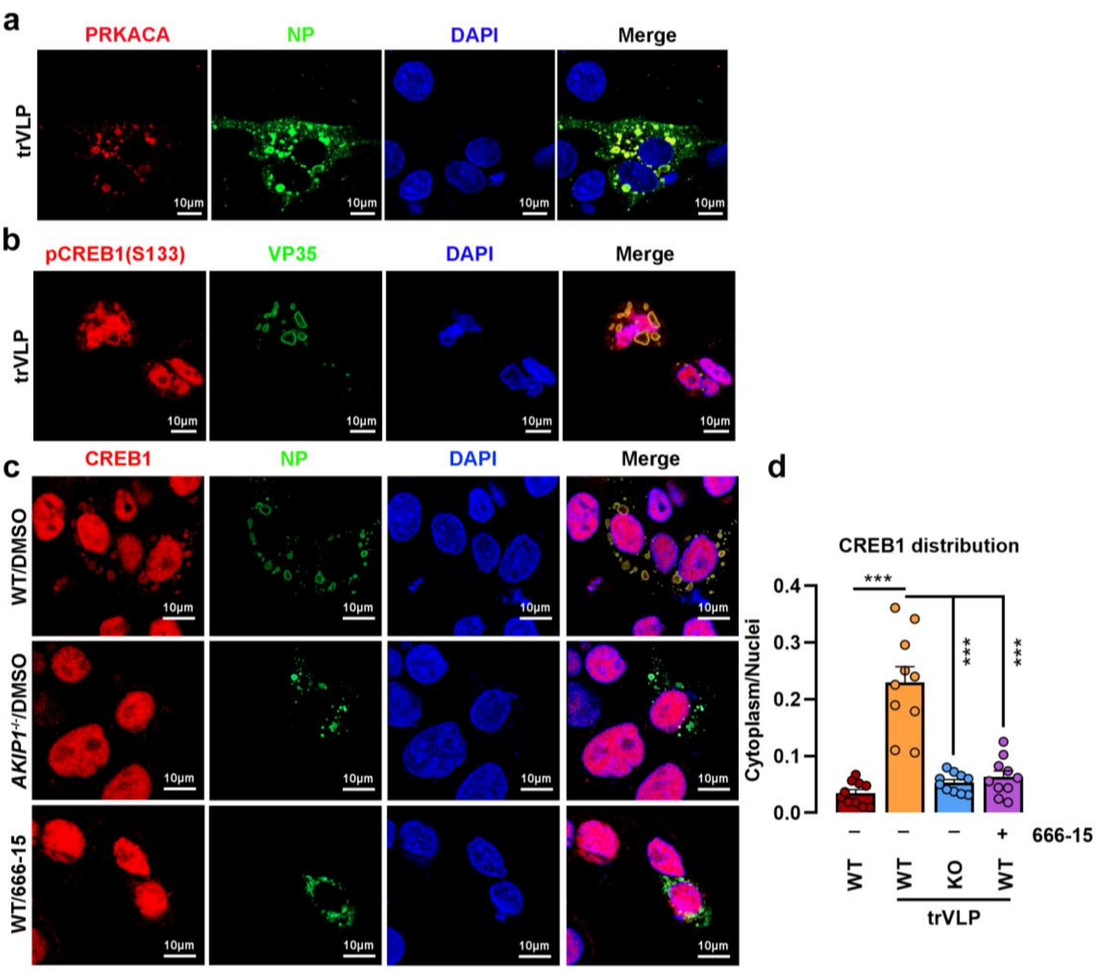

Supplementary Fig. 5 CREB1 is hijacked by viral VP35 into the VIBs of trVLPs-infected cells to regulate viral replication.

a, b, HepG2 cells transfected with the EBOV minigenome (p0) were immunostained with anti-PRKACA (red in **a**), anti-NP (green in **a**), anti-pCREB1 S133 (red in **b**) or anti-VP35 (green in **b**) antibodies. **c, d,** WT and *AKIP1*-depleted (KO) HepG2 cells transfected with the EBOV minigenome (p0) and treated with or without 666-15 (1 μM) were immunostained with the indicated antibodies. The cytoplasmic/nuclear distribution of CREB1 in (**c**) was analyzed using ImageJ software, and the means ± s.e.m. from at least 10 cells are presented (**d**). Differences between the two groups were evaluated using the two-sided unpaired Student's *t*-test. All data from three independent replicates are presented as the means ± s.e.m. (***P < 0.001).

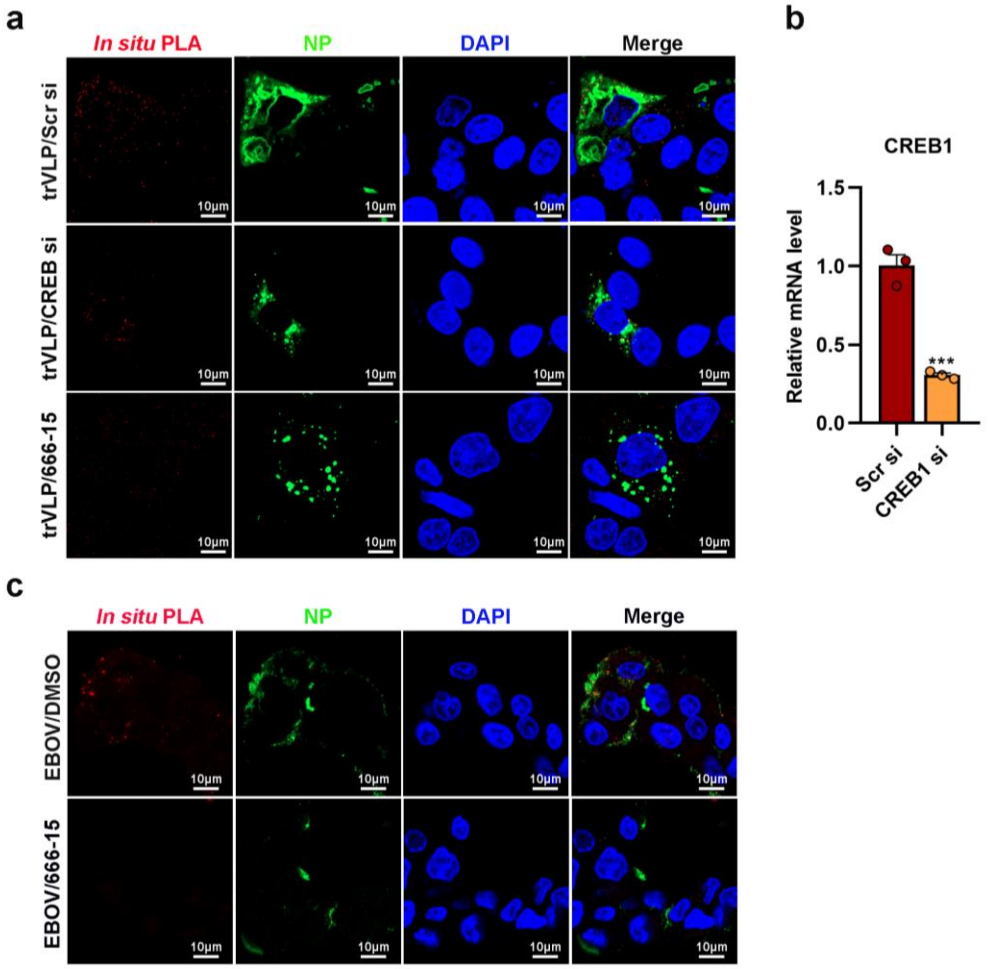

Supplementary Fig. 6 EBOV VP35 and CREB1 association in VIBs after EBOV or trVLPs infection.

a, b, HepG2 cells were transfected with CREB1 siRNA or scrambled (Scr) siRNA for 6 h. Next, the cells were transfected with the EBOV minigenome (p0), treated with or without 1 μM 666-15 for 48 h and then subjected to an *in situ* PLA assay with anti-VP35 and anti-CREB1 antibodies and immunostaining with an anti-NP antibody (green). The silencing efficiency of the CREB1 siRNA was determined using qRT-PCR (**b**). Differences between the two groups were evaluated using the two-sided unpaired Student's *t*-test. The data from three independent replicates are presented as the means ± s.e.m. (***P < 0.001). **c,** HepG2 cells infected with live EBOV (MOI=10) were treated with or without 1 μM 666-15 for 48 h, subjected to an *in situ* PLA assay

with anti-VP35 and anti-CREB1 antibodies, and immunostained with an anti-NP antibody (green). The data from three independent replicates are presented.

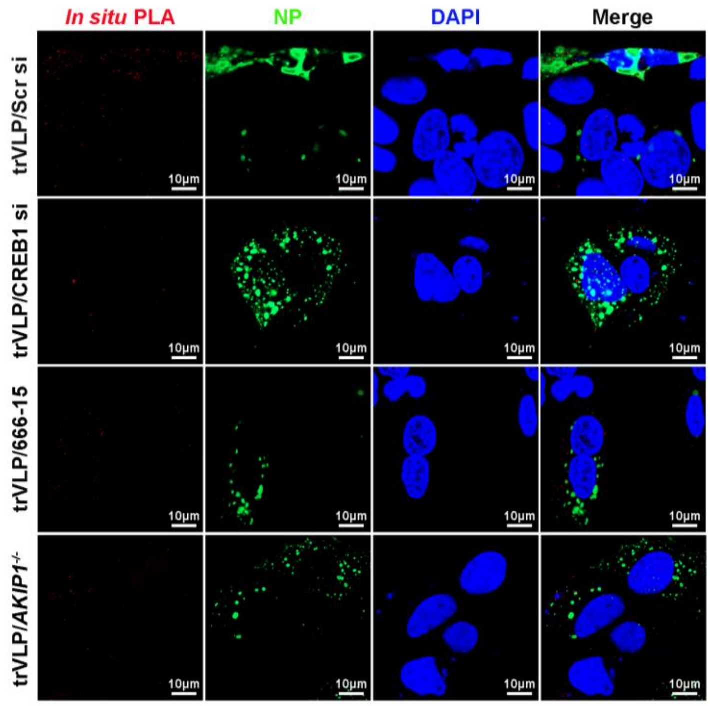

Supplementary Fig. 7 The VP35:L association and VIBs formation are suppressed by *AKIP1* depletion, CREB1 knockdown or CREB1 inhibition.

WT and *AKIP1*$^{-/-}$ HepG2 cells were transfected with the CREB1 siRNA or scrambled (Scr) siRNA for 6 h. Next, the cells were transfected with the EBOV minigenome (p0), treated with or without 1 μM 666-15 for 48 h, subjected to an *in situ* PLA assay with anti-VP35 and anti-L antibodies (red), and immunostained with an anti-NP antibody (green). At least two independent replicates were performed in all of the experiments.

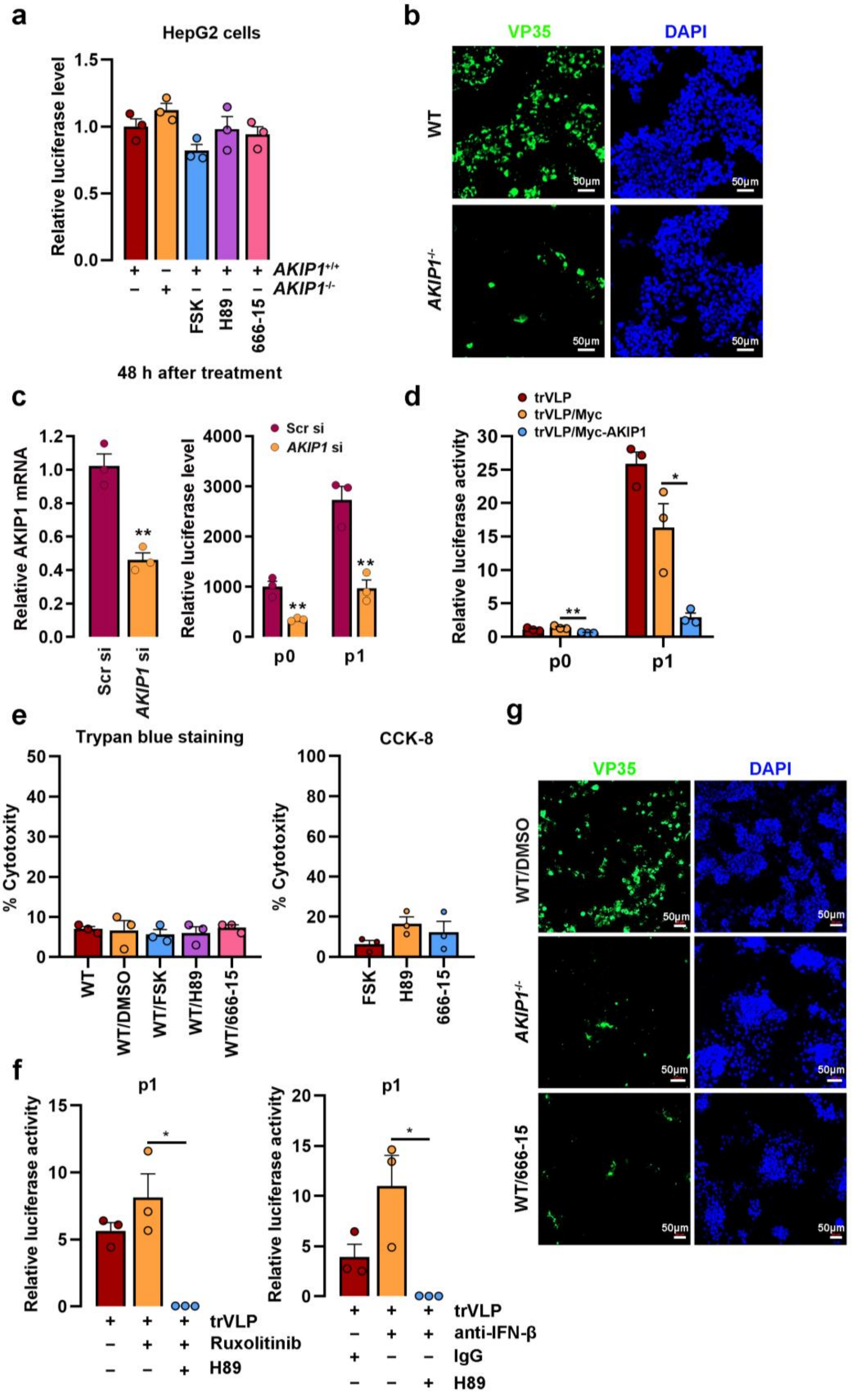

Supplementary Fig. 8 The AKIP1-PKA-CREB1 axis potentiates EBOV replication.

a, WT and *AKIP1*$^{-/-}$ HepG2 cells were transfected with firefly luciferase plasmids and treated with

FSK (25 μM), H89 (10 μM), or 666-15 (1 μM) for 48 h. The firefly luciferase activity was determined. **b,** WT and *AKIP1*$^{-/-}$ HepG2 cells transfected with the EBOV minigenome (p0) for 48 h and then immunostained with an anti-VP35 antibody (green). **c,** HepG2 cells were transfected with the EBOV minigenome (p0 and p1) for 12 h. Then, p1 cells were transfected with the AKIP1 siRNA (*AKIP1* si) or scrambled (Scr) siRNA. The effect of the siRNA on AKIP1 expression was determined using qRT-PCR (left panel), and the amounts of trVLPs were determined using the luciferase activity assay (right panel). Differences between the two groups were evaluated using the two-sided unpaired Student's *t*-test. Data are presented as mean ± s.e.m. (**$P < 0.01$). **d,** HepG2 cells were transfected with Myc vector or Myc-AKIP1 for 6 h. Then, the cells were transfected with the EBOV minigenome. The amounts of trVLPs were determined by luciferase activity assay. Differences between the two groups were evaluated using the two-sided unpaired Student's *t*-test. Data are presented as mean ± s.e.m.(*$P < 0.05$; **$P < 0.01$). **e,** The cytotoxicity of the tested drugs (25 μM FSK; 10 μM H89; 1 μM 666-15) to HepG2 cells was determined by trypan blue staining (left panel) or CCK-8 assays (right panel). **f,** HepG2 cells were transfected with the EBOV minigenome (p1) and treated with an anti-IFN-β antibody (25 ng/ml, left panel), the JAK1 inhibitor ruxolitinib (10 μM, right panel), and/or H89 (10 μM) for 48 h. The amounts of trVLPs were determined using a luciferase activity assay. Differences between the two groups were evaluated using the two-sided unpaired Student's *t*-test. Data are presented as mean ± s.e.m. (*$P < 0.05$). **g,** WT and *AKIP1* KO HepG2 cells infected with live EBOV (MOI=10) were treated with 666-15 (1 μM) or vehicle for 72 h. Cells were then analyzed by immunostaining with an anti-VP35 antibody (green). All data from three independent replicates are presented.

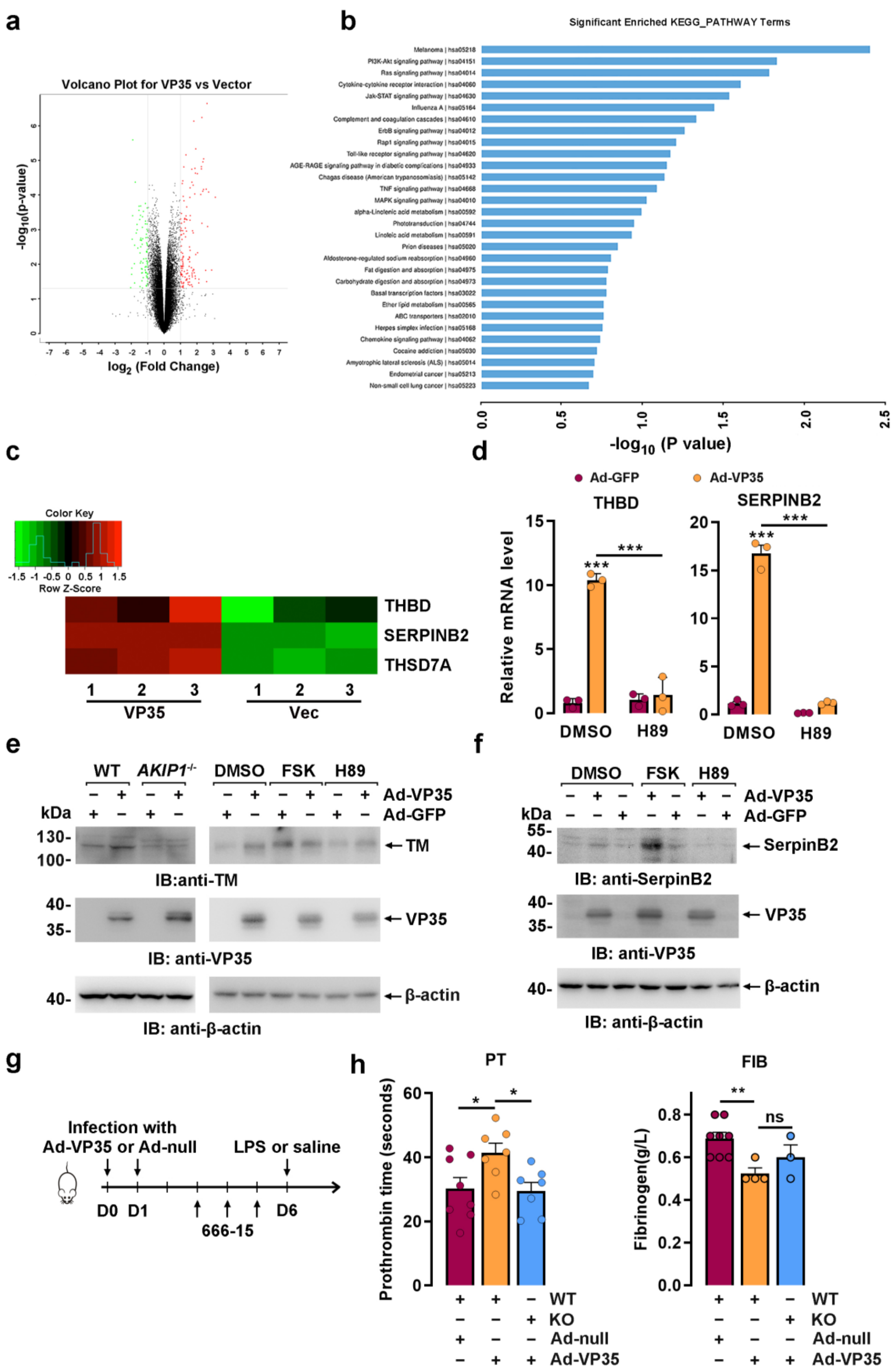

145

146 **Supplementary Fig. 9 EBOV VP35 promotes the transcription and expression of**

147 **coagulation-related genes via the AKIP1-PKA-CREB1 axis.**

a, Gene expression in HepG2 cells transfected with VP35 or vector was analyzed with a gene expression microarray. Differentially expressed genes regulated by VP35 in HepG2 cells are presented via a gene expression spectrum scatter plot. **b,** Top 20 KEGG pathways enriched in differentially expressed genes (≥2-fold or ≤-2-fold) regulated by VP35. **c,** Transcription of coagulation-related genes regulated by VP35 is shown as a heat map. **d,** HepG2 cells infected with Ad-VP35 or Ad-GFP for 48 h were treated with or without H89 (10 μM) for another 4 h. *THBD* and *SERPINB2* mRNA levels were determined by qRT-PCR, and normalized fold induction was demonstrated. Differences between the two groups were evaluated using the two-sided unpaired Student's *t*-test. Data are presented as mean ± s.e.m. (*$P < 0.05$; **$P < 0.01$). **e, f,** HepG2 cells (**e,** left and **f**) or HUVECs (**e**, right) infected with Ad-VP35 or Ad-GFP were treated with FSK (25 μM), H89 (10 μM) or vehicle for 4 h. Cell lysates were analyzed by immunoblotting. **g,** Schematic experimental design of Ad-VP35 or Ad-null infection, as well as 666-15 and LPS administration. **h,** WT and *Akip1*$^{-/-}$ (KO) C57BL/6N mice (10 weeks) were intravenously injected with Ad-VP35 or Ad-null (2×10^9 PFU) via tail vein injection twice at an interval of 24 h. Six days post infection, the mice were injected with LPS (5 mg/kg) via the tail vein. Four hours later, the prothrombin time (PT) and serum fibrinogen (FIB) concentrations were determined (n=3-8). Differences between the two groups were evaluated using the two-sided unpaired Student's *t*-test. The data from three independent experiments are presented as the means ± s.e.m. (ns, not significant; *$P < 0.05$; **$P < 0.01$).

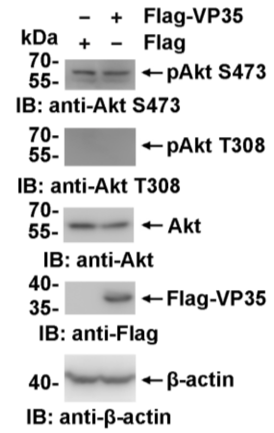

Supplementary Fig. 10 Akt S473 and T308 phosphorylation is not regulated by EBOV VP35.

Lysates of HepG2 cells transfected with Flag or Flag-VP35 plasmid were analyzed by immunoblotting with anti-Akt S473 or anti-Akt T308. The data from two independent experiments are presented.

Supplementary Table

Supplementary Table 1. Primers used for qRT-PCR.

Gene Product	Forward primer (5' to 3')	Reverse primer (5' to 3')
h-GAPDH	AAggTCATCCCTgAgCTgAAC	ACgCCTgCTTCACCACCTTCT
h-AKIP1	AAggCTggCTCTAgAAgTgC	CTgTTTCTCTAggTggggCg
h-CREB1	gTATATTgCCATTACCCAgggAg	CTgCTgCATTggTCATggT
h-THBD	CCCAACACCCAggCTAgCT	gATgTCCgTgCAgATgAAACC
h-SerpinB2	gTTACCCCCATgACTCCAgA	CgCAgACTTCTCACCAAACA
VP40	GGAGGCCATATACCCTGTCAGGTC	GCCTGGTGTGTGGCTGGCAT
3Le	TCTCCgAAgggAgCAAgggCA	ACCCCAAgCTTTAgggTTgTTgAATC
5Tr	TggCCTCTCTCCCTgCgTgA	TCAACCAAAgCACTATTCCATCTggC
h-IFNβ	AGGACAGGATGAACTTTGAC	TGATAGACATTAGCCAGGAG
h-ISG15	CTCTGAGCATCCTGGTGAGGAA	AAGGTCAGCCAGAACAGGTCGT
